# Purification and Characterization of a Thermostable Lipase from *Geobacillus thermodenitrificans* IBRL-nra

**DOI:** 10.1155/2012/987523

**Published:** 2012-11-11

**Authors:** Anuradha Balan, Darah Ibrahim, Rashidah Abdul Rahim, Fatimah Azzahra Ahmad Rashid

**Affiliations:** ^1^Industrial Biotechnology Research Laboratory, School of Biological Sciences, Universiti Sains Malaysia, 11800 Pulau Pinang, Malaysia; ^2^School of Biological Sciences, Universiti Sains Malaysia, 11800 Pulau Pinang, Malaysia

## Abstract

Thermostable lipase from *Geobacillus thermodenitrificans* IBRL-nra was purified and characterized. The production of thermostable lipase from *Geobacillus thermodenitrificans* IBRL-nra was carried out in a shake-flask system at 65°C in cultivation medium containing; glucose 1.0% (w/v); yeast extract 1.25% (w/v); NaCl 0.45% (w/v) olive oil 0.1% (v/v) with agitation of 200 rpm for 24 hours. The extracted extracellular crude thermostable lipase was purified to homogeneity by using ultrafiltration, Heparin-affinity chromatography, and Sephadex G-100 gel-filtration chromatography by 34 times with a final yield of 9%. The molecular weight of the purified enzyme was estimated to be 30 kDa after SDS-PAGE analysis. The optimal temperature for thermostable lipase was 65°C and it retained its initial activity for 3 hours. Thermostable lipase activity was highest at pH 7.0 and stable for 16 hours at this pH at 65°C. Thermostable lipase showed elevated activity when pretreated with BaCl_2_, CaCl_2_, and KCl with 112%, 108%, and 106%, respectively. Lipase hydrolyzed tripalmitin (C16) and olive oil with optimal activity (100%) compared to other substrates.

## 1. Introduction

Lipases (triacylglycerol acylhydrolases, EC 3.1.1.3) catalyse the hydrolysis of long-chain triglycerides with the formation of diacylglycerol, monoacylglycerol, glycerol, and carboxylate, as well as the reverse reaction of the synthesis of esters formed from fatty acids and glycerols [1], present in diverse organisms including animals, plants, fungi, and bacteria. However, only microbial thermostable lipases are commercially significant for their potential use in industries, such as specialty organic syntheses [2], hydrolysis of fats and oils, modification of fats, flavor enhancement in food processing, and chemical analyses [3]. Microbial lipases also have been immensely used for biotechnological applications in dairy, detergents, and textile industries as well as surfactant and oil-processing industries. In fact they have also been widely used in pharmaceutical industries in the production of enaniometrically pure chemicals, since they have a number of unique characteristics couple with in district substrate specificity [4], stable and active in organic solvents [5], do not require cofactors [6], exhibit a high degree of regioselectivity, and possess a wide range of substrate specificity for the conversion of various unnatural substrates [2]. 

Lipases with molecular weight range of 19–60 kDa, belong to the *α*/*β* hydrolase family. The active site is formed by a catalytic triad of Ser, Asp/Glu and His [7]. Lipases share a consensus sequence of GXSXG, whereby X may be any amino acid residue. Lipase exhibits interfacial activation whereby it acts only on emulsified substrates. The active site of lipase is covered by a lid-like *α*-helical structure. The lid moves away upon binding to a lipid interface, causing the active site of lipase fully accessible, enhancing hydrophobic interaction between the enzyme and lipid surface [7].

The major requirement for commercial lipases is thermal stability which would allow enzymatic reaction to be performed at higher temperatures and would be helpful to increase conversion rates, substrate solubility, and to reduce the contamination of microorganism and the viscosity of the reaction medium [1]. This has drawn the interest towards thermophiles in both research and industries. 

Thermostable enzymes are usually derived from thermophilic microbial strains which may be expected to produce intrinsically more heat-stable enzymes than their mesophilic counterparts. Thermophiles growing at the temperature range of 60–100°C have complete thermal equilibrium with the microenvironments and secretes enzymes that are stable at this temperature to support the physiological processes [8]. At present, thermophilic lipases from *Bacillus* sp. are widely studied and this is due to its unique protein sequence and uncommon biochemical properties [9]. Thermophilic *Bacillus* species previously assigned to rRNA group 5 have recently been transferred to a new genus *Geobacillus* [10–12]. The *Geobacillus *species form a phenotypically and phylogeneticallycoherent group of thermophilic bacilli with high levels of 16S rRNA sequence similarity (98.5–99.2%). This group comprises established species of thermophilicbacilli such as *Bacillus stearothermophilus* [13], *Bacillus thermocatenulatus* [3],* Bacillus thermoleovorans* [14], *Bacillus kaustophilus* [15], *Bacillus thermoglucosidasius,* and *Bacillus thermodenitrificans* [11].

*Geobacillus thermodinitrificans* IBRL-nra was originally isolated from a hot spring in Labok, Kelantan, Malaysia with the temperature of 45°C–50°C. Its optimal thermostable lipase production in a 5-L stirred-tank bioreactor was previously reported by Balan et al. 2010 [16]. Therefore, the aim of the present work was to purify and characterize the thermostable lipase produced by this strain.

## 2. Materials and Methods

### 2.1. Microorganism and Culture Maintenance

The bacterial strain, IBRL-nra, used in this study was isolated from a Malaysian hot spring in Labok, Kelantan and identified by 16S rRNA analysis as *Geobacillus thermodenitrificans* [17]. It was cultured on nutrient agar and maintained at 65°C. The strain was subcultured every two weeks to maintain its viability.

### 2.2. Cultivation of Microorganism

*G*. *thermodenitrificans* IBRL-nra was grown in a culture medium consisted of 1.00% (w/v) glucose, 1.25% (w/v) yeast extract, and 0.45% (w/v) NaCl and the pH was adjusted to 6.8. After sterilization, 0.10% of olive oil was added together with the 5.0% (v/v; 5 × 10^6^ cells/mL) of inoculum which was prepared earlier (the inoculum was prepared by transferring 1-2 colonies of *G*. *thermodenitrificans* IBRL-nra into 3 mL of culture medium, incubated at temperature 65°C with agitation 200 rpm for 24 hours). The inoculated culture medium was then incubated at temperature 65°C with agitation 200 rpm for 24 hours.

 The fermentation culture was harvested, filtered and centrifuged at 6000 g for 15 minutes. The cell-free supernatant was collected and used as the crude enzyme.

### 2.3. Lipase Assay

Lipase activity was determined by using the modified colorimetry method of Kumar et al., 2005 [18]. Culture filtrate (1.0 mL) was shaken with 2.5 mL of olive oil emulsion, 1.48 mL of 100 mM phosphate buffer (pH 7.0), and 20 *μ*L of 20 mM CaCl_2_ in an orbital shaker at an agitation speed of 200 rpm for 30 minutes at 65°C. The emulsion was prepared by mixing together 1% polyvinyl alcohol and olive oil (3 : 1; v/v) in a homogenizer. The enzyme reaction in the emulsion system was stopped by adding 6 M HCl (1.0 mL) and isooctane (5.0 mL), followed by mixing using a vortex mixer for 30 s. The upper isooctane layer (4.0 mL) containing the fatty acid was transferred to a test tube containing copper reagent (200 *μ*L) and mixed vigorously. The reagent was prepared by adjusting the solution of 5% (w/v) copper (II) acetate-1-hydrate to pH 6.1 with pyridine. The absorbance of the upper layer was read at 715 nm. Lipase activity was measured by measuring the amount of free fatty acids released based on a standard curve of free fatty acid (oleic acid). One unit of lipase activity was defined as the amount of enzyme releasing 1 *μ*mole of fatty acid per minute

(1) Lipase  activity  (U/mL)  =μmol/mlmin⁡.

### 2.4. Determination of Protein Content

Protein content of cell-free supernatant was determined according to Lowry method [19], using bovine serum albumin as standard.

### 2.5. Purification

The collected extracellular crude lipase was purified using a three step procedures: ultrafiltration, followed by affinity chromatography and gel-filtration chromatography. The crude lipase was concentrated using the ultracentrifugal filter (Milipore-Amicon) with membrane pore size of 3000 Da about 30 times. 

### 2.6. Affinity Chromatography

The concentrated enzyme collected from the previous step was loaded on a HiTrap Heparin column (5.0 mL, 1.6 cm × 2.5 cm) equilibrated with 10 mM phosphate buffer (pH 7.0). The unbound protein was washed out with low ionic strength buffer (10 mM phosphate buffer, pH 7.0) until the protein was undetectable at absorbance 280 nm. Then, the enzyme was eluted with high strength buffer (10 mM phosphate buffer, 1-2 M NaCl, pH 7.0) using a step elution method. The flow rate was adjusted to 16 mL/hour and the fraction volume of 4.0 mL was collected.

### 2.7. Gel-Filtration Chromatography

The fraction containing lipase with highest activity from affinity chromatography was loaded on Sephadex G-100 column (40.0 cm × 1.2 cm) equilibrated with 100 mM phosphate buffer, pH 7.0. The enzyme was then eluted with the same buffer with a flow rate of 1 mL/min. Fractions of 4 mL were collected.

### 2.8. Determination of Molecular Weight

The molecular mass of the purified lipase was determined by SDS-PAGE as described by Laemmli, 1970 [20] using 12.5% acrylamide gel. Unstained standard protein range: *β*-galactosidase (116.0 kDa), bovine serum albumin (66.2 kDa), ovalbumin (45.0 kDa), lactate dehydrogenase (35.0), Rease Bsp981 (25.0 kDa), and *β*-lactoglobulin (18.4 kDa) were used as a molecular weight marker. The gel was stained with silver staining method as described by Bollag et al. 1996 [21].

### 2.9. Effect of Temperature on Enzyme Activity and Stability

 For the optimum temperature determination, lipase activity was measured by colorimetric assay at different temperature in the range of 40–90°C at pH 7.0 in 100 mM phosphate buffer. 

 For the thermostability, the purified lipase was incubated at 60, 65, 70, and 75°C for up to 24 h in 100 mM phosphate buffer, pH 7.0 and residual activity was measured at intervals of 1 h. 

### 2.10. Effect of pH on Enzyme Activity and Stability

 The lipase activity was determined at 65°C in a pH range of 4.5–10.5 using 100 mM different buffers: acetate buffer for pH 4.5, 5.0; phosphate buffer for pH 5.5, 6.0, 6.5, 7.0, and 7.5; Tris-HCl buffer for pH 8.0 and 8.5; glycine-NaOH buffer pH 9.0, 9.5, 10.0, and 10.5.

 For the pH stability, the purified lipase was incubated at pH 6.5, 7.0 and 7.5 in different buffers for 24 h at 65°C. Residual activity was measured at intervals of 1 h.

### 2.11. Effect of Metal Ions on Lipase Activity

The purified lipase was preincubated with each of the selected metal ions at final concentration of 1 mM at 65°C for 30 min prior to lipase assay. The lipase activity of the purified enzyme without metal ions was defined as 100%.

### 2.12. Substrate Specificity

For substrate specificity, triglycerides (C2–C18) with concentration of 10 mM and natural oils (corn oil, palm oil, soy bean oil, canola oil, and sunflower oil) were used as the substrates. The olive oil emulsion was substituted with the various substrates and lipase assay was carried out at 65°C with shaking at 200 rpm for 30 min using the calorimetric method as described earlier.

## 3. Results and Discussion

### 3.1. Microorganisms

**Figure 1:**
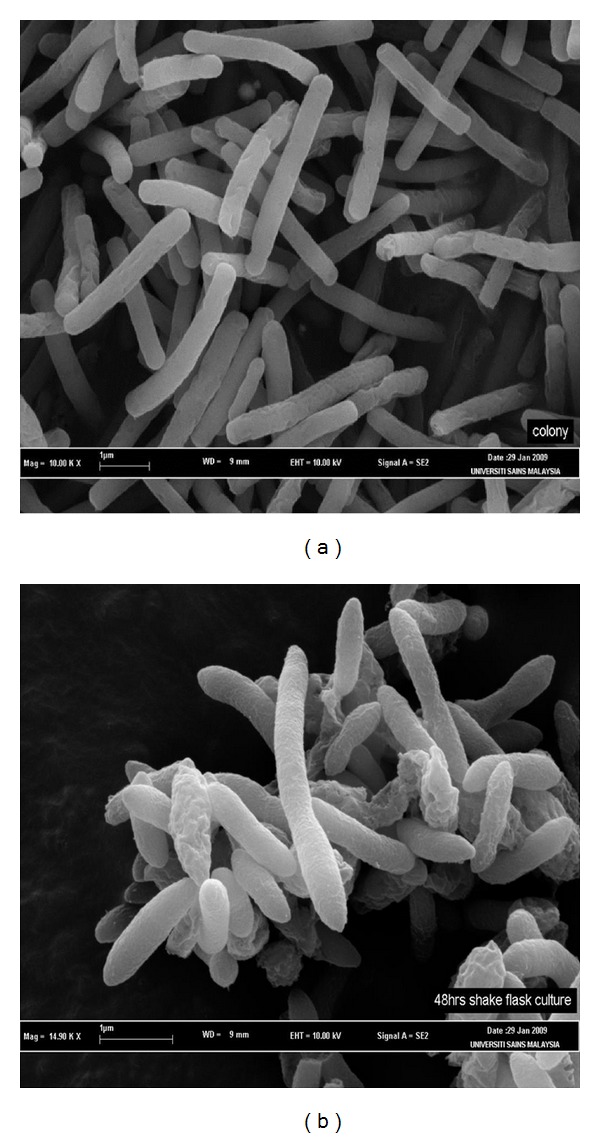
SEM micrograph of *G. thermodenitrificans *IBRL-nra. (a) 24 h old cells that were grown on nutrient agar slant, (b) the 24 h old cells that were grown in a cultivation medium in a shake flasks system.

*Geobacillus thermodenitrificans* IBRL-nra as indicated in the name itself is a heat-loving bacteria which is capable of reducing nitrate to nitrogen. It is a Gram-positive rod which forms flat, lobate and off-white colonies (Figure 1). It can grow at 45–70°C at pH 6–8 in 0.30% NaCl. *G*. *thermodenitrificans* IBRL-nra used in this study was previously isolated from a hot spring in Kelantan, Malaysia [17].  Figure 1(a) shows the 24 h old cells that were grown on nutrient agar slant, whereas Figure 1(b) shows the 24 h old cells that were grown in the cultivation medium. Both micrographs show typical cells of *G*. *thermodenitrificans *IBRL-nra. 

### 3.2. Purification

**Figure 2:**
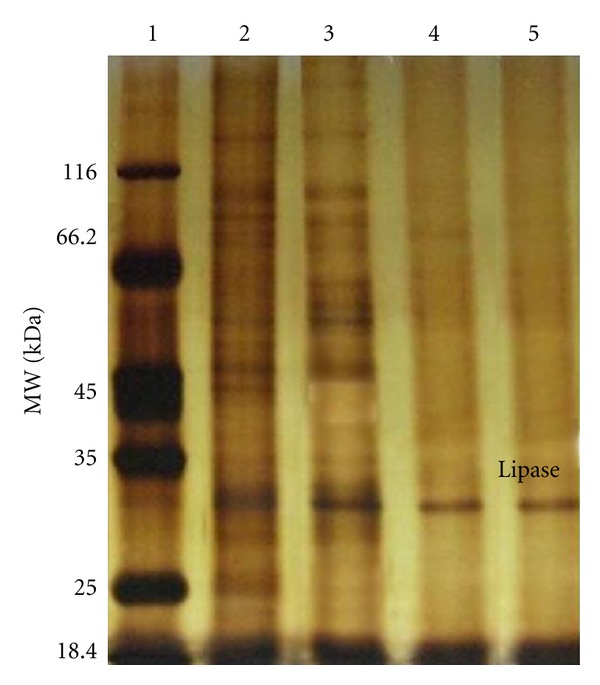
SDS-PAGE (12.5%) of thermostable lipase from *G. thermodenitrificans *IBRL-nra. Lane 1: unstained protein molecular weigth marker are *β*-galactosidase (116.0 kDa), bovine serum albumin (66.2 kDa), ovalbumin (45.0 kDa), lactate dehydrogenase (35.0 kDa), Rease Bsp981 (25.0 kDa), and *β*-lactoglobulin (18.4 kDa). Lane 2: crude lipase. Lane 3: concentrated lipase. Lane 4: partially purified lipase. Lane 5: purified lipase.

 The extracellular lipase from *G*. *thermodentrificans* IBRL-nra was purified using a three step procedures: ultrafiltration, affinity chromatography, and gel filtration chromatography. The highest lipase activity was detected at fraction 18 with 308 U/mL in affinity chromatography. Affinity chromatography was used in this study to minimize the purification steps and the loss of enzyme [6]. Heparin used is a highly sulphated glucosaminoglycan with a broad affinity for lipase. The partially purified lipase was then chromatographed on Sephadex G-100 gel filtration. A single peak of lipase was detected at fraction 17 with 92.2 U/mL activity. SDS-PAGE analysis of lipase exhibited a single-band with molecular mass estimated to be 30 kDa (Figure 2). Lipases from *Bacillus* are reported to have low molecular weight of ~20 kDa [22]. Lipases with lower molecular weight have advantage as smaller enzymes are more stable due to smaller changes (unfolding) in tertiary structure [23]. The purification summary is tabulated in Table 1. After a three step purification procedures, crude lipase was purified to homogeneity by 34 fold from the culture supernatant with specific activity of 36.7 U and a final recovery of 9%. Sifour et al. 2010 [24] reported that a thermostable lipase was purified from *Geobacillus stearothermophilus* using ultrafiltration, Q-Sepharose ion exchange chromatography, Sephadex G-100 gel filtration, and adsorption on hydroxyl apatite to 22.6 fold with 8.8% recovery and molecular weight of 61 kDa. Smaller lipases have been reported by Chartrain et al. 1993 [25] (29 kDa), Kohno et al. 1994 [26] (30 kDa), Ohnishi et al. 1994b [27] (24 kDa), Mase et al. 1995 [28] (24 kDa), Lee et al. 1999 (35 kDa) [29], and Sharma et al. 2002 [30] (37 kDa). Kumar et al., 2005 [18], reported that an alkaline thermostable lipase was purified from *B*. *coagulans *BTS-3 with molecular weight of 31 kDa. In contrast, thermotolerant metallolipase from *B*. *coagulans *MTCC-6375 was reported to be 103 kDa in size [31]. However, there are also relatively higher Mr lipase that have been reported from *B*. *stearothermophilus* [13], *B*. *thermocatenulatus* BTL2 [32], *Bacillus* sp. 398 [33], and *Bacillus* sp. J33 [34] possessing Mr of 62.5 kDa, 69 kDa, 50 kDa, and 60 kDa, respectively.

The final yield of thermostable lipase is quite low, however this study is only to characterize the enzyme for its good properties. The enzyme yield may be enhanced by immobilization or cloning and expression of the lipase gene. Immobilization of lipase enhances its stability and activity and improves recyclability of the enzyme [6]. Palomo et al. 2004 [35] reported that lipase activity of *Bacillus thermocatenulatus* (BTL2) immobilized with hydrophobic resin (octadecly-Sepabeads) was increased and retained 100% of its initial activity after incubation for 50 h at 65°C. 

### 3.3. Effect of Temperature

**Figure 3:**
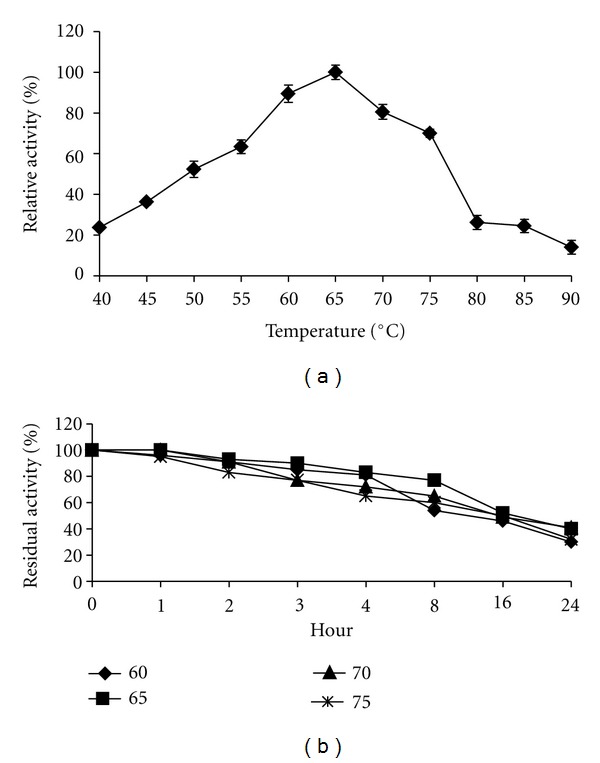
Effect of temperature on thermostable lipase activity. The lipase activity at 65°C (130.56 U/mL) was set as 100% (a). Thermostability of the purified lipase. The activity of lipase without preincubation (95.0 U/mL) was set as 100% (b).

Figure 3 shows the effect of temperature on lipase activity. It was detected in temperature range of 40–90°C (Figure 3(a)) at pH 7.0. The optimum temperature of purified lipase was 65°C, followed by 60 and 70°C. The activity dropped sharply above 75°C with only 20% of activity left at 80°C. Four temperatures (60, 65, 70, and 75°C) with highest activity were then chosen for thermostability study. There was no loss of activity for the first 60 minutes at 65 and 70°C (Figure 3(b)) and the activity dropped slightly thereafter. However, around 90% of the original activity was retained for 3 h and 2 h at 65 and 70°C, respectively. The result obtained shows that the thermostable lipase of *G. thermodenitrificans* IBRL-nra is highly stable compared to lipase from *Geobacillus stearothermophilus *[24] with only 87.5% of its original activity retained after 15 minutes of exposure at 70°C. The half life of the purified lipase was 8 h at 60°C, 16 h at 65, 70, and 75°C, respectively (Figure 3(b)). Sharma et al. 2002 [30] reported that the lipase from *Bacillus *sp. RSJ-1 had optimum activity at 50°C and it retained 96, 92, 78 and 34% of its maximum activity and half-life of 150, 90, 55, and 45 minutes at 55, 60, 65, and 70°C, respectively. Another highly thermostable lipase was isolated by Wang et al., 1995 [36] from *Bacillus* strain and the half life of the enzyme was 8 hour at 75°C and it retained at least 90% of the original activity for 15 hour at 60°C. The characteristics of the lipase from *G. thermodenitrificans IBRL-nra* make it belongs to the thermostable enzyme because it showed an optimal activity at 65°C.

### 3.4. Effect of pH

**Figure 4:**
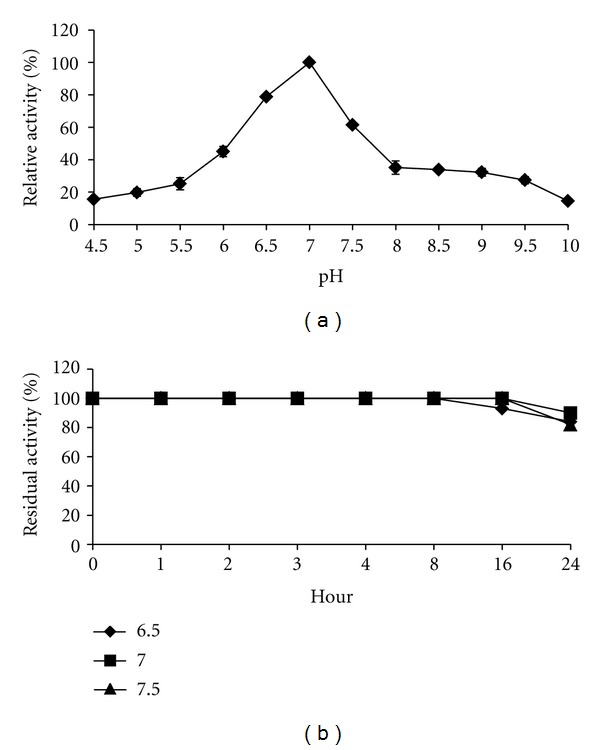
Effect of pH on thermostable lipase activity at 65°C. The lipase activity at pH 7.0 (102.5 U/mL) was set as 100% (a). pH stability of purified thermostable lipase, the activity of thermostable lipase without preincubation (95.0 U/mL) was set as 100% (b).

Changes in pH will affect the protein structure and the enzyme activity [27]. The effect of pH on lipase activity is shown in Figure 4, where lipase showed activity in the pH range of 6.0-8.0 (Figure 4(a)). Maximal activity was observed at pH 7.0 followed by 6.5 and 7.5 (phosphate buffer) and the activity dropped at pH 8.0 onwards. No loss of activity over 16 h was observed when the lipase was preincubated at pH 7.0 and 7.5 (Figure 4(b)). 100% of activity was retained for 8 h at pH 6.5. However lipase retained 85%, 90%, and 80% of its original activity for 24 h at pH 6.5, 7.0, and 7.5, respectively. Purified lipase from *Geobacillus stearothermophilus*, retained 95–100% of its original activity for 30 minutes at 60°C after incubation at pH 5–9 [21]. Kumar et al., 2005 [18] reported that the purified lipase from *B*. *coagulans* BTS-3 was stable within a pH range of 8.0–10.5 with optimum activity at pH 8.5. Lipase from *B*. *stearothermophilus* MC7 had pH optimum within the range of 7.5–9.0 and was stable at alkaline pH range 7.0–11.0 at 60°C [22]. Study on lipase from *Bacillus* sp. RSJ-1 showed a maximum activity at pH 8.0 (100%) and followed by pH 9.0 (99%) and it retained 84% and 82% of its maximum activity at pH 8 and 9, respectively, for 2 hours at 50°C [30]. 

### 3.5. Effect of Metal Ions on Lipase Activity

**Figure 5:**
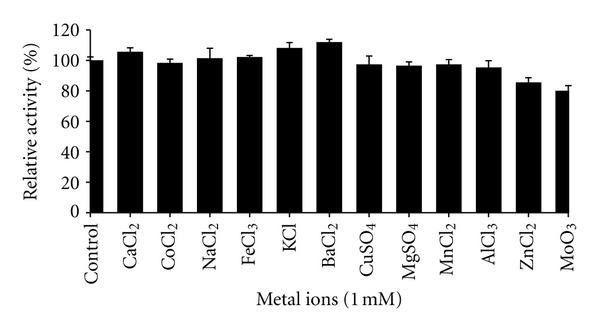
Effect of metal ions on thermostable lipase activity. Lipase activity without addition of metal ions, control (111.7 U/mL) was set as 100%.

Metal ions are reported to stimulate lipase-catalyzed hydrolysis of oil by removing the fatty acids from the oil-water interface and allowing lipase to act freely on oil molecules [23]. Thermostable lipase activity was enhanced by several metal ions (1 mM) with highest relative activity achieved when the enzyme was pretreated with BaCl_2_, CaCl_2_, and KCl with 112%, 108%, and 106%, respectively (Figure 5). Kambourova et al. 2003 [13] suggested that the positive effect of Ca^2+^ is due to formation of insoluble ion-salts of fatty acids during hydrolysis, thus avoiding the product inhibition. Rahman et al. 2005 [37] stated that metal ions will bind to the enzyme and change the enzyme's conformation to counter better stability and hence greater activity.

However the enzyme activity was slightly inhibited to 78% when preincubated with MoO_3_. In a previous study, the thermotolerant lipase from *Bacillus* sp. RN2 was slightly enhanced by KCl, CaCl_2 _and ZnCl_2_ [34]. Sifour et al., 2010 [24] also found CaCl_2_ strongly improved the lipase activity from *Geobacillus stearothermophilus* Strain-5 to 155%. However the lipase activity was inhibited by CuSO_4_ and HgCl_2_. 

### 3.6. Substrate Specificity

**Figure 6:**
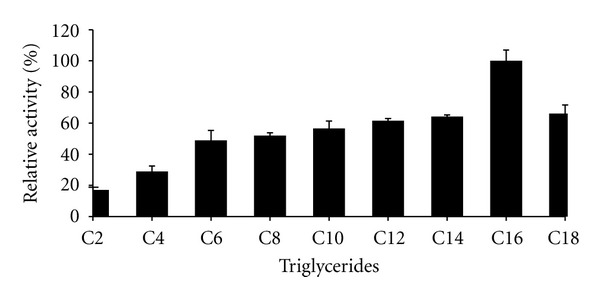
Effect of triglycerides on thermostable lipase activity. Highest lipase activity at C16 (109.18 U/mL) was set as 100%.

**Figure 7:**
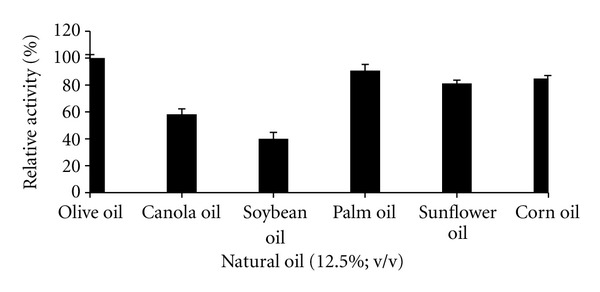
Effect of natural oils on thermostable lipase activity. Lipase activity of 122.29 U/mL using olive oil as the substrate (control) was set as 100%.

 Figures 6 and 7 displays the substrates (triglycerides and natural oil) specificity of purified lipase. Lipase activity increased from C8 to C16 and the highest lipase activity was observed at C16 (tripalmitin) with 100% activity, followed by C18 (tristearin) and C14 (trimystrin) with 60% activity. The enzyme hydrolyzed triacylglycerols with acyl-group chain lengths between C8 and C18 with better activity compared to short chain acyl-group (C2–C6). The preference of the enzyme towards long-chain triacylglycerols indicates that it is a true lipase. Its high affinity towards long-chain triacylglycerols suggests that this enzyme has potential role in the food and diary industry especially in hydrolysis of milk fat, cheese ripening, modification of butter, and flavour development in meat and fish. 

In the natural oil study, lipase hydrolyzed olive oil with optimal activity (100%) followed by palm oil (96%), corn oil (90%), and sunflower oil (86%). The lowest lipase activity was obtained when soybean oil and canola oil were used as the substrate. Lipase from *G. stearothermophilus* Strain-5 had highest affinity towards tributyrin [38], while thermoalkaliphilic lipase of *Geobacillus* sp. T1 hydrolyzed sunflower oil rapidly [39]. The ability of this thermostable enzyme in hydrolysis of vegetable oils could be employed in fat and oil industry especially retailoring and upgrading the vegetable oils into nutritionally important products like PUFA and cocoa butter replacer.

 Based on the results obtained, the thermostable lipase from *G*. *thermodenitrificans *IBRL-nra is a potential candidate for biotechnological application and could be exploited in industries especially in food and dairy, pharmaceutical, nutraceuticals, detergent, fat and oil, and organic synthesis industries. The enzyme also has potential usage in the production of biodiesel from vegetable oils. However the enzyme yield could be further improved via immobillization and cloning to enhance the good properties of this enzyme. The cloning and overexpression of the lipase gene from *G. thermodenitrificans *is in progress and the recombinant thermostable lipase will be used for the three-dimensional structure elucidation.

## Figures and Tables

**Table 1:** Summary of purification of thermostable lipase from *G*. *thermodentrificans*.

Purification step fold	Total activity (U)	Total Protein (mg)	Specific activity (U/mg)	Yield (%)	Fold
Crude lipase	4167.0	3840.0	1.09	100.0	1
Ultrafiltration	3088.82	942.28	3.28	74.1	3.0
Affinity Chromatography	1232.0	144.32	8.54	29.6	7.8
Gel-filtration	368.88	10.05	36.7	8.9	33.7
